# Segmental absence of intestinal musculature in the cecum of an adult identified during endoscopic submucosal dissection

**DOI:** 10.1055/a-2409-0022

**Published:** 2024-09-19

**Authors:** Satoshi Abiko, Kosuke Nagai, Kenji Kinoshita, Kazuteru Hatanaka, Yoshiya Yamamoto, Hirohito Naruse

**Affiliations:** 1 Gastroenterology and Hepatology, Hakodate Municipal Hospital, Hakodate, Japan; 2 378609 Digestive Disease Center, Showa University Koto Toyosu Hospital, Tokyo, Japan

Segmental absence of intestinal musculature (SAIM) is an uncommon cause of spontaneous perforation in newborns 1 and is extremely rare in adults 2 . Recently, cases of SAIM causing perforation during endoscopic submucosal dissection (ESD) of the esophagus and stomach have been reported 3 4 ; however, SAIM has never been reported in the cecum in adults. Herein, we report a case of SAIM in the cecum of an adult that was identified during ESD.

ESD was performed for a 73-year-old man with a lesion extruding from the appendiceal orifice following open appendectomy. Despite not having touched the muscular layer, a tiny absence of the intestinal musculature was encountered during the initial circumferential incision ( Fig. 1 **a** ). As the abdominal findings were normal and his vital signs were stable, resection was prioritized. Very marked fibrosis was observed and submucosal dissection was carefully performed around the base of the residual appendix left after the open appendectomy ( Fig. 1 **b** ). After the lesion had been resected, there was a tiny absence of the intestinal musculature of about 5 mm observed near the base of a residual appendix ( Fig. 2 **a, b** ). The site was closed using a clip ( Fig. 2 **c** ). To further protect the base of the ulcer, it was completely closed with several clips ( Video 1 ). Computed tomography after the procedure revealed a small amount of air behind the peritoneum, but fortunately no free air. The patient was managed conservatively and the length of his hospital stay was 13 days.

**Fig. 1:**
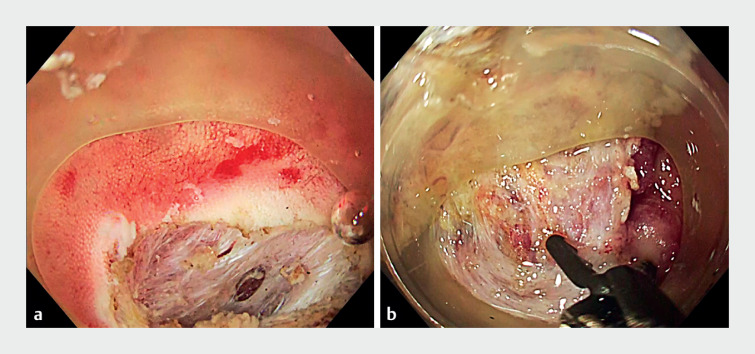
Endoscopic images during the endoscopic submucosal dissection procedure showing: **a** a tiny absence of the intestinal musculature that was encountered during the initial circumferential incision even though we had not touched the muscular layer; **b** very marked fibrosis that required very careful performance of submucosal dissection around the base of a residual appendix.

**Fig. 2:**
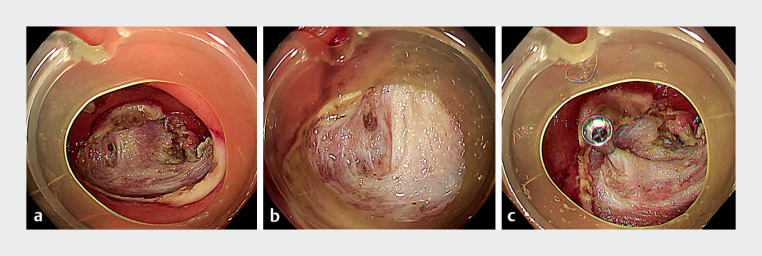
Endoscopic images after endoscopic submucosal dissection of the lesion showing: **a, b** a tiny absence of the intestinal musculature of about 5 mm near the base of a residual appendix; **c** clip closure of the dissection site.

Video showing segmental absence of intestinal musculature in the cecum of an adult that was identified during endoscopic submucosal dissection.Video 1

Acquired SAIM is believed to be caused by ischemia due to multiple surgeries or chronic constipation 5 . In this case, the acquired SAIM may have been a result of the appendicitis and its surgical intervention. When performing cecal ESD near the base of a residual appendix left after open appendectomy, the possibility of SAIM should be kept in mind.

Endoscopy_UCTN_Code_CCL_1AD_2AJ

## Notes

Endoscopy E-Videos https://eref.thieme.de/e-videos
*E-Videos* is an open access online section of the journal *Endoscopy* , reporting on interesting cases and new techniques in gastroenterological endoscopy. All papers include a high-quality video and are published with a Creative Commons CC-BY license. Endoscopy E-Videos qualify for HINARI discounts and waivers and eligibility is automatically checked during the submission process. We grant 100% waivers to articles whose corresponding authors are based in Group A countries and 50% waivers to those who are based in Group B countries as classified by Research4Life (see: https://www.research4life.org/access/eligibility/ ). This section has its own submission website at https://mc.manuscriptcentral.com/e-videos .
